# Course of psychotic experiences and disorders among apprentice traditional health practitioners in rural South Africa: 3-year follow-up study

**DOI:** 10.3389/fpsyt.2022.956003

**Published:** 2022-09-29

**Authors:** Martine C. E. van der Zeijst, Wim Veling, Elliot M. Makhathini, Ndukuzakhe D. Mbatha, Sinethemba S. Shabalala, Daphne van Hoeken, Ezra Susser, Jonathan K. Burns, Hans W. Hoek

**Affiliations:** ^1^Parnassia Psychiatric Institute, The Hague, Netherlands; ^2^Department of Psychiatry, University Medical Center Groningen, Groningen, Netherlands; ^3^Department of Nursing, Durban University of Technology, Pietermaritzburg, South Africa; ^4^Department of Psychiatry, Nelson R. Mandela School of Medicine, University of KwaZulu-Natal, Durban, South Africa; ^5^Department of Epidemiology, Mailman School of Public Health, Columbia University, New York, NY, United States; ^6^New York State Psychiatric Institute, Columbia University Irving Medical Center, New York, NY, United States; ^7^Institute of Health Research, University of Exeter Medical School, University of Exeter, Exeter, United Kingdom

**Keywords:** ancestral calling, apprentice traditional health practitioner, *ukuthwasa*, psychosis, South Africa

## Abstract

**Background:**

Culture is inevitably linked with the experience, interpretation and course of what modern biomedicine understands to be psychotic symptoms. However, data on psychoses in low- and middle-income countries are sparse. Our previous study showed that psychotic and mood-related experiences, symptoms and disorders are common among individuals who had received the ancestral calling to become a traditional health practitioner (THP) in rural KwaZulu-Natal, South Africa. Our related ethnographic study suggested that *ukuthwasa* (the training to become a THP) may positively moderate these calling-related symptoms. As far as we know, no research has been conducted into the course of psychiatric symptoms among apprentice THPs.

**Objective:**

We studied the course of psychotic experiences, symptoms and disorders among apprentice THPs. We also assessed their level of functioning and expanded our knowledge on *ukuthwasa*.

**Materials and methods:**

We performed a 3-year follow-up of a baseline sample of apprentice THPs (*n* = 48). Psychiatric assessments (CAPE, SCAN), assessment of functioning (WHODAS) and a semi-structured qualitative questionnaire were completed for 42 individuals.

**Results:**

At 3-year follow-up, psychotic experiences were associated with significantly less distress and there was a reduction in frequency of psychotic symptoms compared to baseline. The number of participants with psychotic disorders had decreased from 7 (17%) to 4 (10%). Six out of seven participants (86%) with a psychotic disorder at baseline no longer had a psychiatric diagnosis at follow-up. Although the mean level of disability among the (apprentice) THPs corresponded with the 78th percentile found in the general population, 37 participants (88%) reported no or mild disability. Forty-one participants (98%) reported that *ukuthwasa* had positively influenced their psychiatric symptoms.

**Conclusion:**

In rural KwaZulu-Natal, psychotic experiences, symptoms and disorders have a benign course in most individuals who are undergoing the process of becoming a THP. *Ukuthwasa* may be an effective, culturally sanctioned, healing intervention for some selected individuals, potentially because it reframes distressing experiences into positive and highly valued experiences, reduces stigma, and enhances social empowerment and identity construction. This implies that cultural and spiritual interventions can have a positive influence on the course of psychosis.

## Introduction

Studies on the continuum of psychosis in a cross-cultural context have demonstrated the heterogeneity of the experiences depending on the culture (1, 2). The influence of culture is inevitably linked with the experience, interpretation and course of what the biomedical model understands to be psychotic symptoms. Theories on psychosis, however, are biased toward Western, urban, high-income countries, as only a small fraction of research on psychosis has been conducted in low- and middle-income countries (LMICs, see list of abbreviations) (3, 4). Research done in LMICs has been based mainly on persons who visit formal mental health services, while many individuals with mental health problems in LMICs would seek help from traditional health practitioners (THPs) (5–8). The lack of diversity of study populations and research settings means that many relevant contextual variations remain undetected. Therefore, in order to improve our understanding of psychosis, it is essential to examine a wider array of social and cultural contexts (4). In addition, when sociocultural settings are not taken into full account, this may limit the effectiveness of interventions, undermine indigenous knowledge and support systems, and advance the medicalization of social suffering (9–12).

In two prior studies, we emically (13) and etically (14) examined unusual perceptual experiences and other mental disturbances among a specific group of individuals who visited THPs in Vulindlela, a rural area in KwaZulu-Natal, South Africa. Our first, ethnographic, study (13) included interviews with 20 THPs, apprentice THPs, patients and relatives who were visiting THPs, patients with a psychotic diagnosis who were visiting a formal health clinic, and biomedical health practitioners. This study showed that in some individuals hallucinations and other mental disturbances might be explained as part of a certain cultural construct, namely the calling of the ancestors to become a THP^1^. Calling-related mental disturbances are attributed to ancestors who are trying to communicate with a living individual through prophetic dreams and voices. Although the calling is regarded as a gift by THPs, it may manifest as a serious mental illness (13, 15). According to traditional beliefs, the only cure for calling-related illness, which might also involve a range of physical symptoms, is to accept the calling and become a THP by successfully completing an apprenticeship to become a THP (13, 15–17). This process of becoming a THP is called *ukuthwasa*^2^ in isiZulu, the language of the Zulu people (see list of isiZulu terms). During *ukuthwasa*, which is usually followed at the place of a THP-trainer and can take years, individuals who have been ‘diagnosed’ with the ancestral calling attend various ceremonies and rituals where they sing, dance, drum and slaughter animals. In addition, they are treated with traditional medicines. It is the role of the trainer to give detailed explanation and guidance to the apprentices, to teach them how to interpret what the ancestors are saying and to manage the dreams and voices as communication, rather than as distressing symptoms. Later during the process, apprentices assist their trainer while she is seeing patients. They are taught how to convey messages from the ancestors to others, how to conduct rituals and where to find traditional medicines themselves (13, 16, 18). By following the process of *ukuthwasa*, the hallucinations and other mental disturbances are believed to change from seriously disturbing at the onset, to beneficial and positive at the outcome (13, 16). The THPs we interviewed in our ethnographic study (13) reported to be cured from the distress and dysfunction of their mental disturbances during their *ukuthwasa*. They had transformed into well-functioning and respected members of the society with a defined work, role and social status as THPs, and their hallucinatory experiences remained an instrumental part of their healing profession. In our second study (14), we assessed 48 apprentice THPs who were undergoing *ukuthwasa –* individuals who are called *amathwasa –* to see whether and how their calling-related mental disturbances could be interpreted from a Western, psychiatric perspective. Our results showed that psychotic and mood-related experiences, symptoms and disorders were common in this group, confirming suggestions from other studies (19–23) and our own ethnographic study (13) that there is a relationship between the cultural construct of ancestral calling to become a THP and what Western psychiatry would characterize within the context of psychosis. Our data indicated that the psychotic phenomena of the *amathwasa* ranged from subclinical psychotic experiences to clinical psychotic disorder. Furthermore, in combination with our ethnographic study, we found indications that the process of *ukuthwasa* could have a beneficial influence on the course of psychotic symptoms in individuals who respond to this process, potentially because it offers a model embedded in the culture for reducing stigma and promoting recovery (13, 14).

This new study on our cohort of *amathwasa* was designed to gain more knowledge on the heterogeneity of psychosis and to further explore the potential recovery-promoting effects of *ukuthwasa*. As far as we know, there are no reports on the course of the psychiatric experiences and symptoms of *amathwasa* over time, nor on the level of functioning of *amathwasa* and THPs. We conducted the follow-up study 3 years after our baseline assessments. This time interval was based on our qualitative study and the literature, which led us to expect that the majority of the *amathwasa* would have finished *ukuthwasa* and be working as THPs within 3 years.

Our primary aim was to examine the course of psychotic events in *amathwasa*. We assessed the occurrence of their (distressing) psychotic experiences, symptoms and disorders after a follow-up period of 3 years and compared these with our baseline assessments. We also assessed the individual levels of functioning after 3 years. Additionally, we intended to further explore the cultural construct of ancestral calling and the process of *ukuthwasa*.

## Materials and methods

### Baseline study

Full details of the study site and recruitment of the study cohort have been described in Van der Zeijst et al. (13, 14). Briefly, this study was conducted in Vulindlela, a rural area in the Msunduzi Municipality in KwaZulu-Natal, South Africa, with a population of approximately 250,000. Vulindlela is characterized by widespread poverty and unemployment (24). There are nine primary healthcare clinics and the closest psychiatric referral hospital is Town Hill in Pietermaritzburg. In KwaZulu-Natal, there are only enough beds to provide adequate in-patient psychiatric care for 25% of those requiring hospitalization (25). The HIV infection prevalence in this region of KwaZulu-Natal is the highest in the country with 60% in women aged 25–40 years and 40% in men aged 25–40 years (26).

In our initial study, 48 apprentice THPs aged 21–48 years were included between September and December 2013. These individuals were referred to our study by THPs, based on two criteria: (1) they were experiencing the ancestral calling, which was identified by THPs based on the presence of certain signs and symptoms – including powerful and prophetic dreams, and (2) they were undergoing the process of *ukuthwasa* with a THP at the time of referral (14). The THPs who cooperated in our baseline study had participated in an epidemiological pilot study on the incidence, early course and treatment pathways of psychotic disorders in a rural South African setting (FEP-INCET study) (27), of which the current research was an add-on study. For the FEP-INCET study, a group of 50 THPs from Vulindlela had been carefully selected and classified as skilled and trustworthy by a regional Traditional Council. We succeeded in establishing collaboration with these THPs, through building of trust by recognizing and acknowledging local authorities, mutual respect for health constructs, taking time to find common ground, and adaptation of the procedures to sociocultural norms (27). From these 50 THPs, individuals were selected who were training apprentice THPs. After their consent to participate, we requested the THPs to refer all their apprentices to our sub-study, and not to make any selection if they were training more than one apprentice THP. Ultimately, five THPs referred apprentices to the current study (14).

Two separate interviews were conducted. The second interviewers were blind to the results of the first interview. The first interview included the Community Assessment of Psychic Experiences (CAPE) (28) and was administered by local isiZulu-speaking students in psychology. The second interview was a diagnostic interview using the Schedules for Clinical Assessment in Neuropsychiatry (SCAN) (29), and was conducted by three local isiZulu-speaking psychiatrists, who were fluent in both isiZulu and English and familiar with the Zulu culture. The SCAN was limited to sections relevant to mood disorders and psychotic disorders. All the SCAN interviews were reviewed during a consensus meeting of the three interviewers and a senior psychiatrist, and a diagnostic classification according to the Diagnostic and Statistical Manual of Mental Disorders 5th edition (DSM-5) (30) was made for each participant.

### Follow-up study

#### Participants and procedure

**FIGURE 1:**
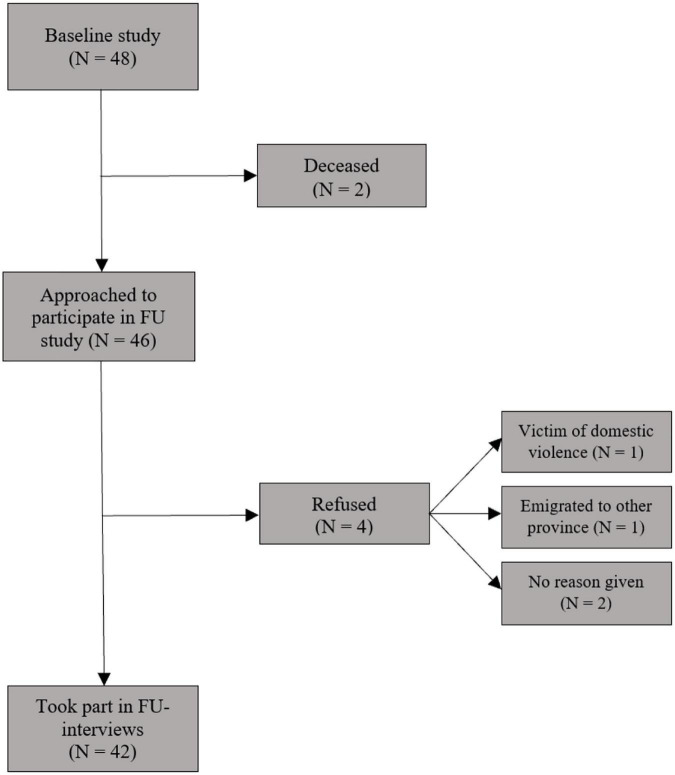
Flowchart for re-recruiting individuals from baseline study into follow-up study.

Three years (mean 39.0 months, SD = 0.7, range 38 – 40 months) after the first contact (14), the apprentice THPs (*n* = 48) were traced and the 46 surviving individuals were invited to participate in our follow-up study. In order to find the individuals and obtain their collaboration, they were first called by an experienced local research assistant together with the first author (MZ) using the phone numbers they had provided at baseline. Non-responders were called again 3 days later. In this way, we reached 15 participants. In order to trace other participants, the project manager (co-author EM), who is also a psychiatric nurse, the research assistant and MZ visited the THPs who had referred the apprentices to our baseline study. With their help we were able to trace the other 33 baseline participants. Two individuals had died; according to the referring THPs one was stabbed to death and one was poisoned. We gave 46 individuals information about our follow-up study and asked them to participate. Four individuals could not participate: one asked not to be contacted due to domestic violence (according to the referring THP), one had emigrated to another province more than 500 km away, and two refused. In total, 42 out of 46 surviving individuals (91%) gave their informed consent and participated in the follow-up study (see Figure 1). They underwent three separate interviews on the same day at Town Hill psychiatric hospital in Pietermaritzburg. The interviews were conducted by three different interviewers who were blinded to the information gathered in the other interviews. Participants received compensation for their time and transportation.

This cohort study, which complements an epidemiological pilot study on the incidence, early course and treatment pathways of psychotic disorders in a rural South African setting (FEP-INCET study) (27), was approved by the regional Zulu *Inkosi* (chief) and his Traditional Council, and by the University of KwaZulu-Natal Biomedical Research Ethics Committee (file number BEO68/11).

#### Measures

At follow-up, the same instruments were used as in the baseline study: the CAPE (28) and SCAN version 2.0 (29). We also added a new instrument, the WHO Disability Assessment Schedule 2.0 (WHODAS 2.0) (31), and a self-designed qualitative questionnaire. The first interview, which was conducted by a trained local research assistant who was a Master student in psychology, included sociodemographic characteristics, the CAPE and the WHODAS 2.0. The second interview included the SCAN and was conducted by two trained local psychiatrists, who were fluent in isiZulu and English, and who were familiar with the Zulu culture. They were the same psychiatrists who had conducted the SCAN at baseline, but were blinded to the baseline SCAN results. The third interview concerned a self-designed semi-structured qualitative questionnaire, and was conducted by the first author (MZ) and co-author EM. Forty interviews were held in isiZulu, and two in English. EM acted as interpreter. Notes were taken constantly and all the qualitative interviews, which lasted 90–150 min, were audio-recorded on tape.

##### Community Assessment of Psychic Experiences

The CAPE (28) is a 42-item questionnaire assessing the positive (20 items), negative (14 items), and depressive (8 items) dimensions of psychotic experiences in the general population. Although this is a self-report instrument, it was administered by a research assistant due to the high level of illiteracy in our research area. As in the baseline study, we used the isiZulu version of the CAPE, which we had previously translated according to the WHO guidelines of forward translation, back-translation and adaptation (14). The CAPE measures both the frequency of, and distress associated with, psychotic experiences. Frequency and distress scores range from 1 (‘never’ or ‘not distressing’) to 4 (‘nearly always’ or ‘very distressing’).

##### Schedules for Clinical Assessment in Neuropsychiatry

The SCAN is a semi-structured diagnostic interview to assess psychiatric symptoms and diagnoses (29). Consistent with the baseline study, we limited the SCAN to sections relevant to mood disorders and psychotic disorders, asking for present-state symptoms (up to 6 weeks retrospectively). In the SCAN, the clinical severity of a symptom is measured by the duration and frequency of the symptom, and by the degree of interference with mental functions (intensity). The procedure of the consensus diagnostic meeting was identical to that of the baseline study (14), except that the senior psychiatrist for the follow-up study was HWH.

##### World Health Organization Disability Assessment Schedule version 2.0

Functioning was measured using the isiZulu translation of the 36-item version of the WHODAS 2.0. This questionnaire measures a patient’s disability in six domains: (1) understanding and communicating (six items), (2) mobility (five items), (3) self-care (four items), (4) getting along with people (five items), (5) household and work activities (each four items), and (6) participation in society (eight items). The WHODAS 2.0 items are rated on a five-point severity scale, from 1 (no disability) to 5 (extreme disability).

##### Qualitative questionnaire

We designed a semi-structured qualitative questionnaire to further explore the participant’s personal experiences in relation to their social functioning, social status, ancestral calling, calling-related symptoms, process of *ukuthwasa*, and being or becoming a THP.

#### Data analysis

Analyses were carried out using IBM SPSS Statistics software version 27. For group characteristics at follow-up, descriptive statistics were used; results were expressed as either the mean ± standard deviation (SD) or as a percentage of the total group. Consistent with the baseline study (14), CAPE dimension and total scores were dichotomized by recoding item scores ‘never’ or ‘sometimes’ (frequency) and ‘not’ or ‘a bit’ (distressing) to 0, and item scores ‘often’ or ‘nearly always’ (frequency) and ‘quite’ or ‘very’ (distressing) to 1. An experience was rated as present if the dichotomized frequency was 1. Distress was rated as present if the dichotomized distress was 1 for symptoms with a ‘present’ experience (dichotomized frequency 1). The positive dimension, measuring positive psychotic experiences, was further divided into three subdimensions: perceptual anomalies (4 items), bizarre experiences (7 items), and delusional ideations (9 items) (32). In order to investigate the course of psychotic experiences, the mean number of endorsed present and distressing psychotic experiences for each (sub)dimension and the total CAPE score were compared between follow-up and baseline using paired samples *t*-tests. A Bonferroni correction for multiple comparisons was applied for the statistical tests of the total score and three main dimensions of the CAPE (positive dimension, negative dimension and depressive dimension) and the three subdimensions of the CAPE positive dimension (perceptual anomalies, bizarre experiences and delusional ideations).

As in the baseline study (14), SCAN diagnoses were divided into five categories for further assessments: (1) schizophrenia spectrum and other psychotic disorder; (2) depressive disorder with psychotic features; (3) depressive disorder without psychotic features; (4) persistent hallucinations without diagnosis; and (5) no hallucinations and no diagnosis. In order to investigate the course of psychotic and mood-related symptoms and disorders, the distributions of participants with these symptoms and disorders according to the SCAN were compared between the two measurement points, using the Wilcoxon signed-ranks test. For interpretation of the statistical tests of the individual SCAN symptoms, a Bonferroni correction was applied.

Since we had no follow-up data on six cases, in the present article the denominator was the number of individuals who participated in the follow-up study (*n* = 42), which is different from that used in the baseline study (*n* = 48). In order to identify potential bias as a result of loss to follow-up, baseline data were compared between cases who did (*n* = 42) and who did not (*n* = 6) participate in the follow-up study as follows: Fisher’s exact test or Fisher- Fisher-Freeman-Halton exact test for categorical data (gender, marital status, education and SCAN diagnoses) and independent *t*-tests for continuous data (age and CAPE total scores).

In order to investigate the level of disability, we scored the WHODAS 2.0 in two ways. First, we calculated the WHODAS 2.0 total scores based on ‘item-response-theory (IRT)-scoring’ (31). IRT-based total scores theoretically range from 0 to 100, with higher scores indicating greater levels of disability in functioning. Second, we calculated the WHODAS 2.0 domain and total scores based on recommendations in the DSM-5-TR, by dividing the sum of their raw underlying item scores by the number of contributing items (33). DSM-5 field trials have shown that these scores are reliable (33). The WHODAS 2.0 DSM-5-TR based domain and total scores could range from 1 (no disability) to 5 (extreme disability). According to DSM-5-TR recommendations (33), the domain and total scores were computed when at least 75% of the underlying items were completed. We compared the item, domain and total scores between three subgroups of participants, namely participants with: (1) no hallucinations and no diagnosis; (2) persistent hallucinations without diagnosis; and (3) a psychiatric disorder, using the Kruskal–Wallis rank-order test. We used the Bonferroni correction for multiple testing. Also, in analogy to the item scores, we categorized the domain and total scores as follows: scores of 2 or lower indicate ‘no to mild’ disability, scores higher than 2 but lower than 4 indicate ‘moderate’ disability, and scores of 4 or higher indicate ‘severe’ disability.

To elucidate the cultural construct of ancestral calling and the process of *ukuthwasa*, we entered data from the semi-structured qualitative interview into SPSS and descriptive statistics were calculated per item.

## Results

In total, 42 participants (60% female) completed the interviews at follow-up. The mean age of the participants at follow-up was 34.2 years (SD = 7.0) and the majority (86%) were single. All but two participants reported a religious affiliation related to Christianity; these were mostly denominations incorporating traditional African rituals and customs (such as Zion and Shembe). Of the 42 participants with follow-up data, 40 (95%) had finished the process of *ukuthwasa* (71% had graduated as THP, 24% were waiting to graduate) and two (5%) were still undergoing *ukuthwasa*. Thirty-nine participants (93%) were working as a THP (according to the participants, an individual is allowed to work as a THP while awaiting graduation if he/she finished *ukuthwasa* successfully and if his/her ancestors have the willingness to assist him/her). For sociodemographic characteristics, see Table 1. No significant differences were found between the 6 drop-outs and the 42 follow-up participants in terms of sociodemographic characteristics, CAPE total scores and SCAN diagnoses at baseline (see Supplementary Table S1).

**TABLE 1:** Sociodemographic characteristics of the *amathwasa* (*n* = 42).

Demographic variables			
		**Mean**	**SD**
Age (years)		34.2	7.0
		***n***	**%**
Gender	Female	25	60
Marital status	Single	36	86
	Married	4	10
	Divorced or widowed	2	5
Education	Elementary school grades 1–6	3	7
	Middle school grades 7–8	6	14
	High school grades 9–11	16	38
	High school grade 12 or graduated	17	41
*Ukuthwasa*	Ongoing	2	5
	Finished, not yet graduated	10	24
	Graduated	30	71
Occupation	THP, no other job	31	74
	THP and other job	8	19
	Other job (not as THP)	2	5
	Unemployed	1	2

### Course of psychotic experiences, psychiatric symptoms and disorders

#### Psychotic experiences on Community Assessment of Psychic Experiences

At follow-up, positive psychotic experiences were reported by 40 participants (95%), as opposed to 38 participants (91%) at baseline (RR = 1.05, 95% CI = 0.93–1.19). The CAPE positive psychotic experiences that were reported most frequently among the study sample were: ‘Believe in the power of witchcraft, voodoo or the occult’ (*n* = 32; 76%), ‘Under the control of some force/power other than yourself’ (*n* = 30; 71%), ‘Destined to be someone very important’ (*n* = 19; 45%) and ‘Hear voices when you are alone’ (*n* = 16; 38%). The most common distressing CAPE psychotic experiences at follow-up were: ‘Believe in the power of witchcraft, voodoo or the occult’ (*n* = 10; 24%), ‘Have only a few hobbies or interests’ (*n* = 10; 24%), ‘Being persecuted in some way’ (*n* = 9; 21%) and ‘Feel pessimistic about everything’ (*n* = 9; 21%).

Forty-one participants (98%) endorsed at least one CAPE psychotic experience of the positive, negative or depressive dimension, as opposed to 39 (93%) at baseline (RR = 1.05, 95% CI = 0.95–1.16). The mean number of psychotic experiences did not differ significantly between baseline and follow-up for (sub)dimensions and total score.

Positive psychotic experiences were associated with distress in 23 participants (55%), compared to 31 (74%) at baseline (RR = 0.74, 95% CI = 0.53–1.03). The mean number of distressing psychotic experiences decreased significantly between baseline and follow-up for the majority of (sub)dimensions, except for unusual perceptual anomalies and depressive experiences. The total score of distressing psychotic experiences decreased from 6.0 experiences at baseline to 3.0 experiences at follow-up (*p* = 0.001). For the CAPE results, see Table 2 (number of endorsed present and distressing items per (sub)domain and total scores) and Supplementary Table S4 (mean distressing presence of CAPE individual items).

**TABLE 2:** Number of endorsed present and distressing items measured with the CAPE at baseline and follow-up (*n* = 42).

Symptom type^a^	Number of endorsed items							
	Present				Distressing presence			
	Baseline	Follow-up	*t*	*P*-value	Baseline	Follow-up	*t*	*P*-value
	Mean (SD)	Mean (SD)			Mean (SD)	Mean (SD)		
Positive dimension (20 items)	5.7 (4.2)	4.7 (4.0)	1.485	0.145	3.1 (2.7)	1.4 (2.0)	3.978	**<0.001**
Perceptual anomalies (4 items)	1.0 (1.2)	1.0 (1.4)	−0.233	0.817	0.4 (0.7)	0.3 (0.8)	1.000	0.323
Bizarre experiences (7 items)	1.4 (1.5)	1.2 (1.2)	0.980	0.333	0.7 (1.0)	0.3 (0.6)	2.795	**0.008**
Delusional ideations (9 items)	3.3 (2.1)	2.6 (1.8)	2.372	0.022	2.0 (1.8)	0.8 (1.0)	4.309	**<0.001**
Negative dimension (14 items)	2.3 (2.2)	1.8 (2.2)	1.185	0.243	1.8 (2.0)	0.8 (1.3)	3.043	**0.004**
Depressive dimension (8 items)	1.3 (1.7)	1.2 (1.7)	0.307	0.760	1.1 (1.7)	0.9 (1.4)	0.973	0.336
Total (42 items)	9.4 (6.8)	7.8 (6.9)	1.426	0.162	6.0 (5.6)	3.0 (3.7)	3.486	**0.001**

^a^Underlying item scores are dichotomized. Symptom rated as present if frequency was ‘often’ or ‘nearly always,’ and distress rated as present if score was ‘quite’ or ‘very’ in combination with frequency at least ‘often.’

Bold font denotes statistical significance after Bonferroni correction, considering *p* < 0.013 as significant for the total score and three CAPE dimensions (*p* < 0.05/4 tests) and *p* < 0.017 as significant for the three subdimensions (*p* < 0.05/3 t-tests).

#### Psychiatric symptoms on Schedules for Clinical Assessment in Neuropsychiatry

The prevalence of psychotic and mood-related symptoms was compared between baseline and follow-up. Nearly all symptoms showed a downward trend after 3 years, with a significant reduction for visual hallucinations (objects/people) (Wilcoxon *Z* = 2.952, *p* = 0.003) (see Supplementary Table S2).

#### Psychiatric diagnoses

In our follow-up study sample, 5 participants (12%) had a DSM-5 diagnosis, of whom 4 (10%) a psychotic disorder, compared to 11 (26%) and 7 (17%) participants at baseline, respectively. Twenty-two participants (52%) had persistent hallucinations without a diagnosis, both at follow-up and at baseline. Fifteen participants (36%) had no hallucinations and no diagnosis at 3-year follow-up, compared to 9 (21%) at baseline.

At follow-up, 8.6% of the participants [3/(42 – 7)] received a new diagnosis of schizophrenia spectrum or another psychotic disorder. Of the four individuals in total with a schizophrenia spectrum or other psychotic disorder at follow-up, two met the criteria for schizophrenia [of which one is possibly a psychotic disorder due to another medical condition (epilepsy)], one met the criteria for schizoaffective disorder, and one had ‘other specified schizophrenia spectrum and other psychotic disorder, no social impairment.’ Thirty participants (71%) had made a shift in diagnostic category from baseline to follow-up, with 10 out of 11 participants (91%) no longer having a psychiatric diagnosis and 4 participants (13%; [4/(42 – 11)]) receiving a DSM-5 diagnosis for the first time (i.e., had no diagnosis at baseline). The changes in distribution in diagnostic categories from baseline to follow-up were not significant [Wilcoxon *Z*: −1.829 (*p* = 0.067)]. See Table 3 for details.

**TABLE 3:** Stability of diagnostic category across a 39-month interval.

		Diagnostic category at follow-up study					
Diagnostic category at baseline study	Total	Schizophrenia spectrum and other psychotic disorders^b^	Depressive disorder with psychotic features	Depressive disorder without psychotic features^e^	Persistent hallucinations without diagnosis	No hallucinations and no diagnosis	Wilcoxon *Z*
							
	*n* (%)	*n* (%)	*n* (%)	*n* (%)	*n* (%)	*n* (%)	
Schizophrenia spectrum and other psychotic disorders^a^	7 (17%)	1 (14%)	0	0	5 (71%)	1 (14%)	−1.829 (*p* = 0.067)
Depressive disorder with psychotic features^c^	1 (2%)	0	0	0	1 (100%)	0	
Depressive disorder without psychotic features^d^	3 (7%)	0	0	0	2 (67%)	1 (33%)	
Persistent hallucinations without diagnosis	22 (52%)	3 (14%)	0	1 (5%)	8 (36%)	10 (45%)	
No hallucinations and no diagnosis	9 (21%)	0	0	0	6 (67%)	3 (33%)	
Total	42	4 (10%)	0	1 (2%)	22 (52%)	15 (36%)	

^a^Includes DSM-5 diagnosis: unspecified schizophrenia spectrum and other psychotic disorders.

^b^Includes DSM-5 diagnoses: schizophrenia, schizoaffective disorder; other specified schizophrenia spectrum and other psychotic disorder.

^c^Includes DSM-5 diagnosis: major depression with psychotic features.

^d^Includes DSM-5 diagnosis: major depressive disorder.

^e^Includes DSM-5 diagnosis: persistent depressive disorder.

We also explored whether there were more DSM-5 diagnoses amongst those participants who were still doing *ukuthwasa*, who had not yet graduated, and/or who were not yet working as THPs, compared to those who were graduated and functioning as THPs. While statistical analysis was not possible due to very small numbers, at face value there did not seem to be any difference between these groups of participants in terms of rates of psychiatric diagnosis.

#### Functioning of participants at follow-up

The mean WHODAS 2.0 IRT-based 36-item total score was 14.7 (SD = 14.1), with a minimum score of 0 and a maximum of 70.8. This mean roughly corresponds with the 78th percentile of the general population, while five participants (12%) had a total score >35, corresponding with the 90th percentile (31).

All mean WHODAS 2.0 DSM-5-TR based domain and total scores were lower than 2.0, indicating no to mild disability (see Table 4). The mean 36-item total score was 1.5 (SD = 0.5). The domains with the highest mean scores were ‘Participation’ (1.9; SD = 0.9) and ‘Cognition’ (1.8; SD = 0.7). Thirty-seven participants (88%) had 36-item total scores in the ‘no to mild’ disability range, five (12%) in the ‘moderate’ disability range and none in the ‘severe’ disability range. None of the between-group Kruskal–Wallis comparisons of the WHODAS 2.0 DSM-5-TR based item, domain and total scores were significant after Bonferroni correction for multiple testing.

**TABLE 4:** WHODAS 2.0 average item, domain and total scores by SCAN group.

	No hallucinations and no (*n* = 15)	Persistent hallucinations without diagnosis (*n* = 22)	Psychiatric diagnosis (*n* = 5)	Total (*n* = 42)	Kruskal–Wallis test		
WHODAS 2.0 average item, domain and total scores	Mean (SD)	Mean (SD)	Mean (SD)	Mean (SD)	K-W H	df	*P*-value
*Domain 1. Cognition*	*1.8 (0.7)*	*1.8 (0.7)*	*1.8 (0.7)*	*1.8 (0.7)*	*0.030*	*2*	*0.985*
Concentrating on doing something (10 min)	1.9 (1.2)	1.7 (1.1)	1.2 (0.4)	1.7 (1.1)	1.614	2	0.446
Remembering to do important things	1.9 (1.2)	2.0 (1.4)	2.4 (1.9)	2.0 (1.4)	0.110	2	0.947
Analyzing and finding solutions to problems	2.1 (1.2)	2.0 (1.2)	2.4 (1.1)	2.1 (1.2)	0.666	2	0.717
Learning a new task	1.8 (1.2)	1.5 (0.9)	1.6 (0.5)	1.6 (1.0)	0.820	2	0.664
Generally understanding what people say	1.3 (0.7)	1.5 (0.9)	1.8 (0.8)	1.5 (0.8)	2.398	2	0.301
Starting and maintaining a conversation	1.8 (1.2)	1.8 (1.3)	1.4 (0.5)	1.8 (1.2)	0.236	2	0.889
*Domain 2. Mobility*	*1.5 (1.0)*	*1.2 (0.2)*	*1.6 (0.4)*	*1.3 (0.6)*	*6.047*	*2*	0.049*
Standing for long periods such as 30 min	2.1 (1.6)	1.5 (1.0)	1.8 (0.4)	1.8 (1.2)	2.140	2	0.343
Standing up from sitting down	1.3 (1.0)	1.2 (0.4)	1.8 (1.3)	1.3 (0.8)	1.841	2	0.398
Moving around inside your home	1.3 (1.0)	1.0 (0.0)	1.0 (0.0)	1.1 (0.6)	3.688	2	0.158
Getting out of your home	1.2 (0.8)	1.0 (0.2)	1.2 (0.4)	1.1 (0.5)	1.364	2	0.506
Walking a long distance such as a kilometer	1.6 (1.2)	1.1 (0.4)	2.0 (1.2)	1.4 (0.9)	5.062	2	0.080
*Domain 3. Self-care*	*1.3 (0.9)*	*1.1 (00.2)*	*1.2 (0.2)*	*1.2 (0.6)*	*4.777*	*2*	*0.092*
Washing your whole body	1.3 (1.0)	1.0 (0.0)	1.0 (0.0)	1.1 (0.6)	3.688	2	0.158
Getting dressed	1.2 (0.8)	1.0 (0.2)	1.0 (0.0)	1.1 (0.5)	0.386	2	0.824
Eating	1.3 (1.0)	1.0 (0.2)	1.4 (0.9)	1.2 (0.7)	1.471	2	0.479
Staying by yourself for a few days	1.4 (1.1)	1.1 (0.3)	1.4 (0.5)	1.2 (0.7)	2.854	2	0.240
Staying by yourself (alone in home) for a day	1.5 (0.9)	1.1 (0.4)	1.0 (0.0)	1.2 (0.6)	2.433	2	0.296
*Domain 4. Getting along with people*	*1.4 (0.6)*	*1.3 (00.5)*	*1.6 (0.9)*	*1.4 (0.6)*	*0.738*	*2*	*0.691*
Dealing with people you do not know	1.5 (0.9)	1.4 (1.1)	1.2 (0.4)	1.4 (0.9)	1.010	2	0.603
Maintaining a friendship	1.5 (0.9)	1.4 (0.8)	1.6 (1.3)	1.4 (0.9)	0.093	2	0.955
Getting along with people close to you	1.4 (0.8)	1.2 (0.7)	2.0 (1.2)	1.4 (0.8)	6.270	2	0.044*
Making new friends	1.2 (0.8)	1.3 (0.9)	1.6 (0.9)	1.3 (0.8)	3.405	2	0.182
Sexual activities	1.5 (1.1)	1.2 (0.8)	1.6 (1.3)	1.4 (0.9)	1.034	2	0.596
*Domain 5*(1). *Household activities*	*1.8 (1.3)*	*1.2 (00.4)*	*1.9 (1.3)^a^*	*1.5 (0.9)^b^*	*3.376*	*2*	*0.185*
Taking care of your household responsibilities	1.7 (1.3)	1.2 (0.7)	2.0 (2.0)^a^	1.5 (1.1)*^b^*	1.335	2	0.513
Doing most important household tasks well	1.9 (1.4)	1.2 (0.7)	2.0 (1.4)^a^	1.5 (1.1)*^b^*	3.691	2	0.158
Getting all household work done that you needed to do	1.9 (1.4)	1.1 (0.3)	1.5 (1.0)^a^	1.4 (1.0)*^b^*	4.057	2	0.132
Getting your household work done as quickly as needed	1.8 (1.4)	1.2 (0.5)	2.3 (1.5)^a^	1.5 (1.1)*^b^*	3.577	2	0.167
*Domain 5*(2). *Work or school activities*	*1.5 (1.1)^c^*	*1.1 (00.3)^d^*	*1.8 (1.4)^e^*	*1.3 (0.8)^f^*	*1.193*	*2*	*0.551*
Taking care of your day-to-day work/school	1.6 (1.2)^c^	1.2 (0.5)^d^	2.3 (2.3)^e^	1.4 (1.0)^f^	1.333	2	0.513
Doing your most important work/school tasks well	1.5 (1.1)^c^	1.1 (0.3)^d^	1.7 (1.2)^e^	1.3 (0.8)^f^	2.342	2	0.310
Getting all the work done that you needed to do	1.4 (1.1)^c^	1.1 (0.3)^d^	1.7 (1.2)^e^	1.3 (0.8)^f^	1.346	2	0.510
Getting your work done as quickly as needed	1.5 (1.1)^c^	1.2 (0.4)^d^	1.7 (1.2)^e^	1.3 (0.8)^f^	1.311	2	0.519
*Domain 6. Participation*	*1.8 (0.8)*	*1.8 (00.9)*	*2.5 (0.9)*	*1.9 (0.9)*	*2.822*	*2*	*0.244*
Joining in community activities same way as anyone else	1.9 (1.4)	1.7 (1.2)	2.0 (1.7)	1.8 (1.3)	0.292	2	0.864
Problem because of barriers/hindrances around you	2.2 (1.4)	2.0 (1.3)	2.6 (1.8)	2.1 (1.4)	0.621	2	0.733
Living with dignity because of attitudes/actions of others	1.7 (1.0)	2.0 (1.3)	2.2 (0.8)	1.9 (1.1)	1.238	2	0.538
Time spent on health condition or its consequences	1.9 (1.2)	1.9 (1.2)	3.6 (1.1)	2.1 (1.3)	7.042	2	0.030*
Emotionally affected by your health condition	1.8 (1.2)	1.9 (1.4)	2.2 (1.1)	1.9 (1.3)	1.530	2	0.465
Drain on the financial resources of you or your family	1.7 (1.3)	1.7 (1.3)	2.8 (1.6)	1.8 (1.3)	4.361	2	0.113
Problems of your family because of your health problems	1.8 (1.4)	1.8 (1.4)	3.0 (1.9)	1.9 (1.5)	4.737	2	0.094
Doing things by yourself for relaxation or pleasure	1.5 (1.1)	1.7 (1.3)	1.4 (0.5)	1.6 (1.2)	0.448	2	0.799
*Total disability score 36 items*	*1.6 (0.7)*	*1.4 (00.3)*	*1.8 (0.6)*	*1.5 (0.5)*	*2.049*	*2*	*0.359*

^a^*n* = 4; 1 missing.

^b^*n* = 41; 1 missing.

^c^*n* = 14; 1 missing.

^d^*n* = 19; 3 missing.

^e^*n* = 3; 2 missing.

^f^*n* = 36; 6 missing.

*Not significant after Bonferroni correction.

### Qualitative data on ancestral calling and *ukuthwasa*

Nearly all participants (*n* = 41, 98%) reported better wellbeing 3 years after their first assessment. Thirty-five participants (83%) said that their calling-related symptoms had negatively influenced their daily functioning before they started *ukuthwasa*, and that they had a negative attitude toward the calling when they just found out that they were supposed to become a THP, often because this was not the future they had planned for themselves. To the question: ‘Do you consider *ukuthwasa* as a training, a treatment, or both a training and a treatment, and why?,’ 34 participants (81%) reported *ukuthwasa* had acted as treatment at least partially. The two most common reasons reported by the participants were that they experienced illness and that they were treated with traditional medicines when they started *ukuthwasa*. Moreover, nearly all (*n* = 41, 98%) were convinced that *ukuthwasa* had influenced their initial symptoms in a positive manner. For further details, see Table 5.

**TABLE 5:** Selected questions from qualitative interview.

Questions from qualitative interview (*n* = 42)	*n*	%
Wellbeing compared to 3 years ago		
Better	41	98
Same	1	2
Ever experienced mental illness (yes)	7	17
Ever received biomedical treatment for mental illness (yes)	2	5
Experienced calling (yes)	42	100
Consider calling as illness		
Yes	16	38
No	20	48
No, but before *ukuthwasa*: yes	6	14
What signs/symptoms did your calling start with?		
Dreams/visions	19	45
Voices	6	14
Dreams and voices	1	2
Physical and voices	1	2
Physical symptoms	7	17
Other psychological disturbances	3	7
Other	5	12
Influence of symptoms on daily functioning (*n* = 41)		
No influence	6	14
Negative influence	35	83
Initial attitude toward calling		
Positive	3	7
Neutral	4	10
Negative	35	83
Understanding of *ukuthwasa*		
Training	8	19
Treatment	2	5
Both	32	76
Perceived influence of *ukuthwasa* on symptoms		
Positive	41	98
Neutral	1	2
Expected outcome without *ukuthwasa*		
Deterioration of symptoms	12	29
Death	20	48
Deterioration of symptoms or death	10	24
Opinion about traditional healers before calling		
Positive	17	41
Neutral	11	26
Negative	14	33
Diviners in family (yes)	29	69
Current use of *umuthi* (traditional medicine) (yes) *n* = 41	38	91
Substance use		
None	11	26
Snuff (smokeless tobacco)	19	45
Alcohol	1	2
Snuff and dagga	2	5
Snuff and alcohol	4	10
Other combination	5	12

## Discussion

Three years after the first assessments, 95% (*n* = 40) of the participants for whom follow-up data were available (42 out of 48) had completed the process of *ukuthwasa* and 93% (*n* = 39) were working as a THP. Regarding our primary aim (to examine the course of psychotic events in individuals who are undergoing *ukuthwasa*), our four main findings were that, at follow-up: (1) psychotic experiences as measured with the CAPE were associated with significantly less distress than at baseline, although the rates of psychotic experiences remained consistently high; (2) the frequency of psychotic and mood-related symptoms on the SCAN showed a downward trend from baseline, with a significant reduction in the symptoms of visual hallucinations; (3) the number of participants with a psychotic disorder decreased from 7 (17%) at baseline to 4 (10%) at follow-up; and (4) although the overall level of disability among the (apprentice) THPs corresponded with the 78th percentile found in general population studies, 88% reported no or mild disability. Regarding our intention to further explore the cultural construct of ancestral calling and the process of *ukuthwasa*, our most important finding was that nearly all the participants said that *ukuthwasa* had positively influenced their calling-related symptoms.

These findings hold two implications: first, the high rates of psychotic experiences, symptoms and disorders seen at follow-up complement our previous study (14), confirming that there is a relationship between what traditional Zulu’s indicate as ancestral calling and what traditional Western psychiatry understands within the context of psychosis, with the reported psychotic phenomena ranging from subclinical psychotic experiences to clinical psychotic disorder. At follow-up, 41 participants (98%) of the study sample reported psychotic experiences, 22 (52%) had persistent hallucinations and 4 (10%) had a psychotic disorder. In addition, 3 of 35 participants (8.6%) with no psychotic disorder evident at baseline were diagnosed with one at follow-up.

Second, the results provide some support for the theory that the process of *ukuthwasa* has a positive influence on the course of recovery from psychotic phenomena in this special group of people. While there was no significant change in frequency of CAPE psychotic experiences from baseline to follow-up, there was a significant reduction of distress related to these psychotic experiences. These results are congruent with the findings of our qualitative study (13) and a qualitative study of Bakow and Low (16), which describe that *ukuthwasa* seems to be successful in the sense that (apprentice) THPs no longer experience distress, while the hallucinatory experiences may remain as part of the healing (divining) profession. The current study also showed reductions in psychotic symptoms and disorders as measured with the SCAN. Of the seven participants who were diagnosed with an unspecified schizophrenia spectrum disorder at baseline, only one (14%) still had a schizophrenia spectrum disorder 3 years later, namely ‘other specified schizophrenia spectrum and other psychotic disorder – without social impairment.’ The other six participants (86%) no longer had a psychiatric diagnosis. While our sample was small, these outcomes are better than what would be expected based on literature reports: a meta-analysis (34) showed that after an average follow-up period of 4.5 years, 36% of initial cases with unspecified schizophrenia spectrum disorder retained the initial diagnosis, and that 31% of cases with unspecified schizophrenia spectrum disorder shifted toward schizophrenia.

In addition, of the 22 individuals with ‘persistent hallucinations without diagnosis’ at baseline, 3 (14%) shifted into a schizophrenia spectrum or other psychotic disorder. This is lower than the 22% reported for a 3-year meta-analytic risk of psychosis onset in a group of individuals with a clinical high-risk state for psychosis (CHR-P) (35). Ten out of twenty-two (45%) had shifted from ‘persistent hallucinations without a diagnosis’ to ‘no hallucinations and no diagnosis.’ This is a slightly higher proportion than the 35.4% of individuals in remission from an initial CHR-P state, as found in a meta-analysis by Simon et al. (36). This positive symptom course in these individuals who followed *ukuthwasa* is further supported by the findings of our qualitative interview, which showed that 41 participants (98%) had reported an improved wellbeing and a beneficial influence of the process of *ukuthwasa* on their initial symptoms.

Conversely, six out of nine participants (67%) with ‘no hallucinations and no diagnosis’ at baseline had persistent hallucinations at follow-up. This is much higher than the annual incidence of 2.5% (37) and lifetime prevalence of 5.2% (38) of psychotic experiences in the general population. Moreover, 4 out of 22 (18%) previously undiagnosed participants with persistent hallucinations now had a psychiatric diagnosis. One possible explanation is that this specific group of people is very sensitive to developing mental health problems, psychotic experiences in particular. Mature THPs select individuals on the basis of the presence of various perceptual disturbances and other signs and symptoms, which they recognize as the ancestral calling. From our ethnographic study (13) as well as from the qualitative findings of the current study, we learned that the order of occurrence and the combination of symptoms which are part of this ‘calling syndrome’ vary between individuals. Hence, psychotic experiences might evolve over time. Another explanation might be that *ukuthwasa* is ineffective in some apprentice THPs (13), or that *ukuthwasa* has side effects, just like psychological treatments. A systematic review and meta-analysis (39) reported that psychological treatments that were applied as early intervention to prevent psychosis, are associated with significant side effects, with about 10% of participants in such treatments deteriorating, potentially due to increased stigma. In our research area, however, stigma is more related to mental illness than to the ancestral calling and the process of becoming a THP (13). The majority of the participants (83%) had a negative attitude toward the ancestral calling to become a THP when they discovered that they were being called, often because the calling and the process of becoming a THP have a major impact on an individual’s life. While most individuals accept their calling after a while, it is possible that some individuals might continue to feel resistance, even during *ukuthwasa*. Others might experience pressure to live up to the calling and to complete *ukuthwasa* successfully. Although we have not found any evidence for this in the qualitative interviews of our present and ethnographic study (13), it is conceivable that such struggles might have negative psychological effects on some apprentice THPs. Another potential negative effect of *ukuthwasa*, as being a traditional intervention for some individuals with psychotic phenomena, may be a delay in accessing formal mental health care, resulting in poorer outcomes of severe mental disorders like schizophrenia (5).

One of the questions from our previous study (14) was whether the *amathwasa* are individuals with CHR-P, especially in regard to the 22 participants (52%) of our baseline group with ‘persistent hallucinations without diagnosis.’ We found intriguing similarities in characteristics between individuals who experience the ancestral calling and the CHR-P group as described by a recent review (35). At the onset of their ancestral calling, individuals are typically young, present with psychotic symptoms, and have associated impairments in their global functioning, social functioning and quality of life. Furthermore, before they visited a THP, they had often sought help at clinics where their mental disturbances remained undetected (13).

There is robust evidence to show that individuals with psychotic disorders and those at high risk of psychosis have disability (40–42). Also psychotic experiences, regardless of whether mental disorders are present, are often associated with functional impairment (43, 44). Our group of young THPs had a point prevalence of positive psychotic experiences (95%) that is approximately 16 times higher than the lifetime prevalence of psychotic experiences in the general population (5.8%) (38). Although mean total disability scores in our sample were around the 78th percentile of the general population, 37 participants (88%) reported no or mild disability, 5 (12%) reported moderate disability and none reported severe disability. In comparison with a European sample of 245 help-seeking individuals with a clinical high risk state of psychosis (45), the young THPs appear to have lower mean disability scores on all six WHODAS 2.0 domains. For example, on the domain ‘Participation’ (the domain with the highest disability rate of our participants), our sample had a mean score of 1.9; indicating mild disability. The European CHR-P group had mean scores of 2.31 and 2.71 for those who did not and for those who did make the transition to psychosis, respectively, indicating moderate disability (45). Our data suggest that, in this specific group of individuals, having hallucinations does not negatively affect daily functioning *per se*; as individuals with ‘persistent hallucinations without diagnosis’ appear to have comparable levels of impairment than individuals with ‘no hallucinations and no diagnosis.’ This seems to make sense in view of the fact that THPs are expected to communicate with their ancestors, often in the form of hearing voices, as part of their divining function as healers.

More broadly, these findings offer support for the theory that the process of *ukuthwasa* may be a culturally sanctioned intervention that moderates psychosis. At present, we can only speculate on how *ukuthwasa* could influence the course of psychotic events in these selected individuals in this particular culture. Several underlying mechanisms could be considered and they are not mutually exclusive. One possibility is that *ukuthwasa* reduces feelings of stigma and discrimination by enhancing positive identification with THPs and other apprentices, as well as by removing individuals with calling-related mental disturbances from a surrounding in which mental illness might be stigmatized, as they usually move out of their own environment and move in with a THP during *ukuthwasa*. A recent review (46) has shown that stigma is associated with a poor outcome for individuals who are at risk of psychosis, including a higher risk of transition to psychosis. Another possibility is that *ukuthwasa* increases social support and access to normalizing explanations for their perceptual disturbances, in which their negative, distressing experiences are reframed to positive and highly valued experiences in line with local beliefs and practices. The social network of THPs and other *amathwasa* has a normalizing function, thus preventing transition into psychosis. In addition, the training to become a THP leads to a new, constructive role, which reduces the probability of unemployment, social exclusion, poverty and gender inequality – all known to be risk factors associated with psychosis (14, 47). *Ukuthwasa* also contains elements that help the *amathwasa* build up their identity (13, 48). It might be an intervention strategy to ameliorate identity processes, which is seen as a critical aspect of the recovery process during the early stages of psychosis (49).

### Strengths and limitations

One major strength of our study is that we were able to trace 100% of the original cohort (*n* = 48) and had a response rate of 91% in the follow-up assessments (*n* = 42, excluding two deaths). This high rate was surprising, especially given the rural and poor nature of the research area where electricity and prepaid calling credit cannot be taken for granted and where people tend to change their mobile phone numbers relatively often, and given the fact that the participants moved away from the homes of the THPs to their own homes after completing *ukuthwasa*. Another strength is that we had extra information provided by the qualitative interviews, which helped us to interpret the quantitative results. The psychiatric assessments were conducted by the same local and isiZulu-speaking psychiatrists and psychologist who performed the baseline study, therefore reducing possible biases. Although the psychiatrists were blind to the baseline SCAN results, we cannot completely rule out the possibility that they might have remembered certain participants, even though follow-up is more than 3 years later.

Most of the limitations in this follow-up study are related to the methodology and data interpretation of the baseline study (14), such as the relatively small study sample. As a consequence of our small sample size, our study may suffer from false-positive results, and at the same time fail to detect meaningful small to medium effects. We therefore encourage replications of our study. The *amathwasa* were included through THPs who also contributed to the FEP-INCET study (27). In the FEP-INCET study, the research team made a lot of effort to involve all THPs in the research area, which enhances the representativeness. Regarding the baseline study of *amathwasa*, the THPs were specifically asked to refer all their apprentices, and not to make any selection (14). Nevertheless, since we do not know how many *amathwasa* stayed at THPs in the research area, we cannot be certain how representative our study sample is. Furthermore, we used the isiZulu version of the CAPE and the WHODAS. These instruments have been translated from English to isiZulu according to WHO guidelines, which includes forward and back-translation. Although back-translation is a process of validity checking (50), the isiZulu version of the CAPE and WHODAS have not been fully validated. Related to this: the full version of the CAPE, which we used in the baseline and follow-up study, includes the item: ‘Do you believe in the power of witchcraft, voodoo or the occult?’ As it is not uncommon for people in our research area to believe in witchcraft and the occult, a positive score on this item cannot simply be regarded as a delusional ideation. Therefore, the CAPE results (Table 2) should be interpreted with caution. At follow-up, the item ‘Do you believe in the power of witchcraft, voodoo or the occult?’ was reported as present (at least often) by 76% of the *amathwasa* and causing distress (at least quite) in 24%, compared to 67% and 43% at baseline, respectively. Supplementary Table S3 shows the outcomes of the respective (sub)dimension and total score when the item about witchcraft was excluded from our calculations.

In the follow-up study, six participants were lost to follow-up. Two had died (due to violence), one was a victim of domestic violence, one moved to another province, and two baseline participants refused to take part in the follow-up study. Because there were no significant differences between the 6 drop-outs and 42 follow-up participants in terms of sociodemographic characteristics, CAPE total scores and SCAN diagnoses at baseline, it seems unlikely that selection bias due to losing six participants have impacted our results in a significant way.

Another limitation is that we have no clear view of substance abuse among our participants, nor of the exact effects of traditional medicines on the mental state of the apprentice THPs. Furthermore, we know from the qualitative interviews that 39 participants (93%) underwent traditional treatment to cleanse them from *indiki*: male spirits of African origin that are not of the same patrilineage as the individual (51). *Indiki* treatment was conducted some time before or directly prior to the process of *ukuthwasa* and, according to many participants, this process also had a positive influence on some of their mental disturbances. However, we have no clear understanding of the role that *indiki* treatment plays in relation to the course of mental disturbances among apprentice THPs.

While there is growing evidence which shows that traditional treatments conducted by THPs are either innocuous or beneficial in the field of mental health (52, 53), there are also reports which show that some forms of traditional health approaches include treatment methods that are potentially harmful, or that detract from due attention to the human rights of patients with serious mental disorders (52, 54). Therefore, we devoted a lot of time and effort to ensure positive collaboration and patient safety with a selected group of THPs in the region. The THPs involved in the current study participated in our FEP-INCET study (27), for which they had been carefully selected and classified as bona fide by a regional Traditional Council. These THPs were all well-known by our project coordinator (third author, EM), who is both a psychiatric nurse and has worked closely with traditional leadership and THP groups in the region for more than 30 years. Furthermore, the THPs involved in our study have a code of conduct guiding their practice. Also, the THPs are recognized by the regional and provincial departments of the Ministry of Health, which had organized a number of workshops for the THPs. In 2018 a meeting took place at which THPs, local biomedical healthcare providers, staff members of the Ministry of Health and our research team came together to discuss the findings of our study. Here, the discussed work of the THPs and our collaboration with them was supported by all stakeholders. Finally, in none of our studies have we received evidence of or observed such harmful practices by these THPs, including the FEP-INCET study and our ethnographic study – which was conducted at the places of THPs – nor were such harmful practices reported by the apprentice THPs in the extensive qualitative interviews of the follow-up study.

Lastly, in the current study, we continued to explore the calling illness from a psychiatric (universalistic) perspective. We would like to emphasize, however, that reducing the experience to a neurobiological disorder does not adequately address its complexity. For a full understanding of the calling illness, as of all mental illnesses in a global context, it is essential to adopt a culture-specific, pluralistic perspective.

## Conclusion

This is the first study to follow the course of mental disturbances and psychiatric diagnoses among *amathwasa* in South Africa, as well as to measure disabilities among them. Our results confirm that, in the context of rural KwaZulu-Natal, the ancestral calling as a cultural construct presents with symptoms that Western biomedicine would typically understand in the context of psychosis, ranging from subclinical psychotic experiences to psychotic disorders. While psychotic experiences and symptoms are very common in most (apprentice) THPs, the majority of THPs and apprentices who are further along in the process of *ukuthwasa*, are well-functioning individuals who do not meet the diagnostic criteria for a psychiatric disorder – even if these experiences and symptoms are associated with distress at times. Especially in a non-Western context, the traditional Western biomedical model may be too narrow for understanding and treating psychosis. The relatively benign course of psychosis in *amathwasa* adds to the theory that the process of *ukuthwasa* may be regarded as an effective, culturally sanctioned, healing intervention for some selected individuals. *Ukuthwasa* seems to moderate mental disturbances like psychotic symptoms such that they become positively valued and meaningful features of a new role as THP (13), and might even contribute to reducing the risk of transition into psychosis in most cases. This implies that cultural and spiritual interventions, which often involve elements aimed at reducing stigma and enhancing social empowerment and identity construction, may positively influence the course of psychosis. The field of psychiatry could learn from these approaches, since effective interventions to prevent psychosis or ameliorate any other outcome in CHR-P individuals are still much needed (35). Advancing knowledge on factors that modulate the onset of psychosis can inform the development of potentially preventive interventions (55).

Our findings also have broader implications and suggest that in some settings, collaboration between biomedical mental health practitioners and THPs could be effective for the understanding and treatment of psychosis and other mental illnesses, especially in LMICs where access to public healthcare is one of the greatest challenges today (6, 56). Although there are still major hurdles to overcome for both biomedical practitioners and THPs in order to work together in the field of mental health (6, 56), formal integration of THPs into public health systems could offer an effective way of addressing both a large treatment gap and the limited resources available, resulting in health and cost benefits.

## Data availability statement

The raw data supporting the conclusions of this article will be made available by the authors, without undue reservation, to any qualified researcher.

## Ethics statement

The studies involving human participants were reviewed and approved by University of KwaZulu-Natal Biomedical Research Ethics Committee (file number: BEO68/11). The patients/participants provided their written informed consent to participate in this study.

## Author contributions

MZ, HH, WV, ES, and JB contributed to the study conception and design. MZ, EM, NM, SS, and HH contributed to the acquisition of data. MZ and DH contributed to the statistical analysis. MZ, DH, HH, and WV contributed to the interpretation of data. MZ contributed to the drafting of manuscript. MZ, HH, WV, ES, JB, DH, and EM contributed to the critical revision. All authors read and approved the submitted manuscript.

## Footnotes

1 The ancestral calling mainly applies to one of the three subtypes of traditional health practitioners, namely to the *isangoma* (diviner). The other two subtypes include the *inyanga* (herbalist) and *umthandazi* (faith healer), who usually have not received a calling. For readability, we here use the umbrella term ‘traditional health practitioners,’ while understanding that this term is heuristic as traditions are always changing.

2 It should be noted that in the literature the term *ukuthwasa* is often used to refer to the illness associated with the ancestral calling to become a THP. However, all our study participants used *ukuthwasa* to refer to the process of becoming a THP, and not to refer to the calling and/or associated illness. Therefore, when we use *ukuthwasa* in this article, we mean to indicate the process of becoming a THP.

## Abbreviations

CAPE: Community Assessment of Psychic Experiences
CHR-P: Clinical High-Risk state for Psychosis
DSM-5: Diagnostic and Statistical Manual of Mental Disorders, 5th edition
FEP-INCET: First Episode Psychosis – INCidence rate, Early course and Treatment pathways
LMICs: Low and Middle Income Countries
SCAN: Schedules for Clinical Assessment in Neuropsychiatry
THP: Traditional Health Practitioner
WHODAS 2.0: World Health Organization Disability Assessment Schedule version 2.0
*Amathwasa*: individuals with the ancestral calling who are undergoing *ukuthwasa* as apprentice traditional health practitioners
*Indiki*: male spirits of African origin that are not of the same patrilineage as the individual
IsiZulu: the language of the Zulu
*Umuthi*: traditional medicine used by traditional healers for healing purposes
*Ukuthwasa*: the process of becoming a traditional healer, usually a diviner.
