# Supplementary material for: Course of psychotic experiences and disorders among apprentice traditional health practitioners in rural South Africa: 3-year follow-up study
Source: Front Psychiatry. 2022 Sep 29;13:956003. doi: 10.3389/fpsyt.2022.956003 (PMC9558832; doi:10.3389/fpsyt.2022.956003)
Supplement: Supplementary file 1 [file Table_1.docx]

**TABLE S1.** Baseline characteristics of drop-outs vs. participants who completed the follow-up study.

|  | **Drop-outs**  **(n=6)** | **Completers of FU**  **(n=42)** | **Statistical test** | |
| --- | --- | --- | --- | --- |
|  | **Mean (SD)** | **Mean (SD)** | **t (df)** | **p-value** |
| Age | 28.3 (7.6) | 30.4 (7.2) | -0.663 (46) | 0.511 |
| CAPE total scores  Total frequency score  Total distress score | 5.5 (3.5)  3.8 (2.8) | 9.4 (6.8)  6.0 (5.6) | -2.176 (11.4)  -0.924 (46) | 0.052  0.360 |
|  | **n (col %)** | **n (col %)** | **χ^2^** | **p-value** |
| Gender  Male  Female | 2 (33)  4 (67) | 17 (40)  25 (60) | NA^a^ | 1.000 |
| Marital status  Single  Married  Widowed | 5 (83)  0 (0)  1 (17) | 35 (83)  7 (17)  0 (0) | 4.838^b^ | 0.155 |
| Education  Elementary school grades 1-6  Middle school grades 7-8  High school grades 9-11  High school grade 12 or graduated | 1 (17)  0 (0)  1 (17)  4 (67) | 3 (7)  5 (12)  19 (45)  15 (36) | 5.795^b^ | 0.575 |
| SCAN diagnosis  No diagnosis  Persistent hallucinations, no diagnosis  Mood disorder  Psychotic disorder | 2 (33)  4 (67)  0 (0)  0 (0) | 9 (21)  22 (52)  4 (10)  7 (17) | 1.414^b^ | 0.825 |

^a^ Fisher’s exact test; two-sided

^b^ Fisher-Freeman-Halton exact test; two-sided
