# Supplementary material for: Course of psychotic experiences and disorders among apprentice traditional health practitioners in rural South Africa: 3-year follow-up study
Source: Front Psychiatry. 2022 Sep 29;13:956003. doi: 10.3389/fpsyt.2022.956003 (PMC9558832; doi:10.3389/fpsyt.2022.956003)
Supplement: Supplementary file 2 [file Table_2.docx]

**TABLE S2.** Frequency distribution of psychotic and mood-related symptoms identified on the SCAN for baseline and follow-up (n=42).

|  | **Baseline** | | |  | **Follow-up** | | |  |  |
| --- | --- | --- | --- | --- | --- | --- | --- | --- | --- |
|  | **Uncommon/ transitory (rarely/every week)** | **Multiple occasions/ part of the time (most days)** | **More or less continuously (nearly all the time)** |  | **Uncommon/ transitory (rarely/every week)** | **Multiple occasions/ part of the time (most days)** | **More or less continuously (nearly all the time)** | **Wilcoxon *Z*** | **p-value** |
|  | **%** | **%** | **%** |  | **%** | **%** | **%** |  |  |
| *Hallucinations* |  |  |  |  |  |  |  |  |  |
| Verbal auditory ^a†^ | 64 | 10 | 0 |  | 36 | 14 | 0 | 1.200 | 0.230 |
| Non-verbal auditory | 29 | 14 | 0 |  | 14 | 12 | 0 | 1.295 | 0.195 |
| Visual, objects/people | 26 | 10 | 0 |  | 7 | 2 | 0 | 2.952 | **0.003** |
| Voices commenting^aǂ^ | 10 | 2 | 0 |  | 7 | 2 | 0 | 0.246 | 0.806 |
| Visual, scenes | 7 | 5 | 0 |  | 14 | 10 | 0 | -1.187 | 0.235 |
| Hypnagogic/hypnopompic | 5 | 0 | 0 |  | 10 | 0 | 0 | -0.816 | 0.414 |
| Olfactory | 7 | 0 | 0 |  | 2 | 0 | 0 | 1.000 | 0.317 |
| Visual, unformed | 0 | 7 | 0 |  | 7 | 0 | 0 | 0.965 | 0.335 |
| Dissociative | 0 | 0 | 2 |  | 5 | 0 | 0 | 0.000 | 1.000 |
| Other senses^b^ | 0 | 0 | 0 |  | 2 | 0 | 0 | -1.000 | 0.317 |
| *Delusions* |  |  |  |  |  |  |  |  |  |
| Being spied upon | 14 | 0 | 0 |  | 2 | 0 | 0 | 2.236 | 0.025 |
| Reference | 10 | 0 | 0 |  | 2 | 2 | 0 | 0.447 | 0.655 |
| Persecution | 10 | 0 | 0 |  | 0 | 0 | 0 | 2.000 | 0.046 |
| Grandiose ability | 7 | 0 | 0 |  | 0 | 2 | 0 | 0.378 | 0.705 |
| Thoughts being read | 2 | 2 | 0 |  | 0 | 0 | 0 | 1.342 | 0.180 |
| Thought insertion | 0 | 0 | 0 |  | 2 | 0 | 0 | -1.000 | 0.317 |

**[TABLE S2. Continued]**

|  | **Baseline** | | |  | **Follow-up** | | |  | |  | |
| --- | --- | --- | --- | --- | --- | --- | --- | --- | --- | --- | --- |
|  | **Minor degree^c^ (little or no loss)** | **Moderate severity, most of period (marked loss)** | **Severe, most of period (total loss)** |  | **Minor degree^c^ (little or no loss)** | **Moderate severity, most of period (marked loss)** | **Severe, most of period (total loss)** | **Wilcoxon *Z*** | | **p-value** | |
|  | **%** | **%** | **%** |  | **%** | **%** | **%** |  |  | |  |
| *Mood-related symptoms* |  |  |  |  |  |  |  |  |  | |  |
| Loss of capacity for enjoyment^a§^ | 10 | 5 | 0 |  | 5 | 5 | 0 | -1.598 | 0.110 | |  |
| Tearfulness and crying | 29 | 5 | 0 |  | 7 | 10 | 0 | 0.894 | 0.371 | |  |
| Depressed mood | 21 | 10 | 0 |  | 0 | 12 | 0 | 1.027 | 0.305 | |  |
| Social withdrawal | 14 | 5 | 0 |  | 0 | 7 | 0 | 0.549 | 0.583 | |  |
| Irritable mood | 12 | 5 | 0 |  | 0 | 0 | 0 | 2.460 | 0.014 | |  |
| Preoccupation with death/catastrophe | 14 | 2 | 0 |  | 2 | 2 | 0 | 1.890 | 0.059 | |  |
| Expansive/elevated mood | 10 | 2 | 0 |  | 0 | 0 | 0 | 2.121 | 0.034 | |  |
| Loss of hope | 7 | 10 | 0 |  | 2 | 0 | 5 | 0.620 | 0.535 | |  |
| Loss of self-esteem | 7 | 2 | 0 |  | 0 | 5 | 2 | -0.412 | 0.680 | |  |
| Self-reported overactivity | 7 | 2 | 0 |  | 0 | 0 | 0 | 1.890 | 0.059 | |  |
| Pathological guilt | 5 | 5 | 0 |  | 2 | 2 | 0 | 0.756 | 0.450 | |  |
| Over-talkativeness | 5 | 2 | 0 |  | 0 | 0 | 0 | 1.633 | 0.102 | |  |
| Distractibility | 5 | 0 | 0 |  | 0 | 0 | 0 | 1.414 | 0.157 | |  |
| Teadium vitae | 5 | 0 | 0 |  | 0 | 2 | 0 | 0.000 | 1.000 | |  |
| Loss of feeling | 2 | 2 | 0 |  | 0 | 0 | 2 | 0.000 | 1.000 | |  |
| Loss of self-confidence | 2 | 2 | 0 |  | 0 | 2 | 0 | 0.272 | 0.785 | |  |

^a^ Items of SCAN using a different scale, converted as follows to present the symptoms in uniformity:

^†^‘rarely’ and ‘every week’ > ‘transitory’; ‘most days’ > ‘part of the time’; ‘nearly all the time’ > ‘continuously’.

^‡^‘voices do comment occasionally’ > ‘transitory’; ‘frequent comments’ > ‘part of the time’.

^§^‘little or no loss, but does not positively enjoy life’ > ‘minor degree’; ‘marked loss’ > ‘moderate severity, most of period’; ‘total loss’ > ‘severe, most of period’.

^b^ Including: sensations of touch, temperature, pain and floating.

^c^ According to SCAN, this is a positive rating of presence, but presence to such a minor degree that it is not appropriate for use in diagnostic classification.

Conspiracy, delusions of grandiose identity, delusions concerning appearance, delusion of depersonalization or realization, thought insertion, thought commentary, sexual delusions, hypochondriacal delusions and familiar people impersonated were not reported by the study group and therefore not included in the table.

Bold font denotes statistical significance after Bonferroni correction, considering p < 0.005 as significant for hallucinations (p < 0.05/10 tests), p < 0.008 for delusions (p < 0.05/6 tests) and p < 0.003 for mood-related symptoms (p < 0.05/16 tests).
