# Supplementary material for: Course of psychotic experiences and disorders among apprentice traditional health practitioners in rural South Africa: 3-year follow-up study
Source: Front Psychiatry. 2022 Sep 29;13:956003. doi: 10.3389/fpsyt.2022.956003 (PMC9558832; doi:10.3389/fpsyt.2022.956003)
Supplement: Supplementary file 3 [file Table_3.docx]

**TABLE S3.** Number of endorsed present and distressing items as measured with the CAPE at baseline and follow-up when the item about witchcraft is excluded (n=42).

|  | **No. of endorsed items** | | | | | | | | |
| --- | --- | --- | --- | --- | --- | --- | --- | --- | --- |
| **Symptom type^a^** | **Present** | |  |  |  | **Distressing presence** | |  |  |
|  | **Baseline** | **Follow-up** |  |  |  | **Baseline** | **Follow-up** |  |  |
|  | **Mean (SD)** | **Mean (SD)** | **t** | ***p-*value** |  | **Mean (SD)** | **Mean (SD)** | **t** | ***p-*value** |
| Positive dimension (19 items) | 5.0 (4.0) | 4.0 (3.8) | 1.682 | 0.100 |  | 2.6 (2.6) | 1.1 (1.9) | 3.603 | **<0.001** |
| Delusional ideations (8 items) | 2.7 (1.9) | 1.8 (1.6) | 3.015 | **0.004** |  | 1.5 (1.6) | 0.6 (0.8) | 4.036 | **<0.001** |
| Total (41 items) | 8.7 (6.6) | 7.0 (6.8) | 1.541 | 0.131 |  | 5.6 (5.5) | 2.8 (3.6) | 3.297 | **0.002** |

^a^ Underlying item scores are dichotomized. Symptom rated as present if frequency was ‘often’ or ‘nearly always’, and

distress rated as present if score was ‘quite’ or ‘very’ in combination with frequency at least ‘often’.

Bold font denotes statistical significance after Bonferroni correction, considering p < 0.017 as significant (p < 0.05/3 t-tests).
