# Supplementary material for: Course of psychotic experiences and disorders among apprentice traditional health practitioners in rural South Africa: 3-year follow-up study
Source: Front Psychiatry. 2022 Sep 29;13:956003. doi: 10.3389/fpsyt.2022.956003 (PMC9558832; doi:10.3389/fpsyt.2022.956003)
Supplement: Supplementary file 4 [file Table_4.docx]

**TABLE S4.** Mean distressing presence of CAPE items, baseline versus follow-up (n=42).

|  | | Distressing presence  (distress at least a bit) | |  | |  |
| --- | --- | --- | --- | --- | --- | --- |
| CAPE questions per (sub)dimension | | **Baseline** | **Follow-up** |  |  | |
|  | | **Mean (SD)** | **Mean (SD)** | **Wilcoxon Z** | **p-value** | |
| Positive experiences | | | | | |  |
| *Perceptual anomalies* | |  |  |  |  | |
| 33 | Hear voices when you are alone | 1.0 (1.2) | 0.5 (0.9) | -2.384 | 0.017 | |
| 34 | Hear voices talking to each other when you are alone | 0.7 (1.1) | 0.4 (0.9) | -1.537 | 0.124 | |
| 41 | A double has taken the place of a family member, friend or acquaintance | 0.7 (1.1) | 0.1 (0.5) | -2.801 | 0.005 | |
| 42 | See objects, people or animals that other people cannot see | 1.2 (1.2) | 0.5 (0.9) | -2.786 | 0.005 | |
| *Bizarre experiences* | |  |  |  |  | |
| 5 | Things in magazines or on TV were written especially for you | 0.7 (1.1) | 0.2 (0.6) | -2.868 | 0.004 | |
| 17 | Electrical devices such as computers can influence the way you think | 0.1 (0.4) | 0.0 (0.0) | -1.890 | 0.059 | |
| 24 | Thoughts in your head are being taken away from you | 0.9 (1.2) | 0.1 (0.3) | -3.674 | **<0.001** | |
| 26 | Thoughts in your head are not your own | 0.8 (1.1) | 0.3 (0.7) | -2.333 | 0.020 | |
| 28 | Thoughts so vivid that you worried other people would hear them | 0.7 (1.0) | 0.1 (0.3) | -3.378 | **<0.001** | |
| 30 | Hear your own thoughts being echoed back to you | 0.7 (1.1) | 0.2 (0.4) | -2.691 | 0.007 | |
| 31 | Under the control of some force/power other than yourself | 1.1 (1.2) | 0.6 (1.0) | -2.250 | 0.024 | |
| *Delusional ideations* | |  |  |  |  | |
| 2 | People seem to drop hints about you or say things with a double meaning | 0.9 (1.2) | 0.5 (0.8) | -1.909 | 0.056 | |
| 6 | Some people are not what they seem to be | 1.0 (1.2) | 0.3 (0.8) | -3.506 | **<0.001** | |
| 7 | Being persecuted in some way | 1.9 (1.2) | 0.9 (1.1) | -3.494 | **<0.001** | |
| 10 | Conspiracy against you | 1.5 (1.2) | 0.7 (1.0) | -3.629 | **<0.001** | |
| 11 | Destined to be someone very important | 0.8 (1.1) | 0.3 (0.7) | -2.158 | 0.031 | |
| 13 | You are a very special or unusual person | 0.6 (1.0) | 0.2 (0.6) | -2.033 | 0.042 | |
| 15 | Communicate telepathically | 0.4 (0.8) | 0.2 (0.5) | -1.563 | 0.118 | |
| 20 | Believe in the power of witchcraft, voodoo or the occult | 1.7 (1.3) | 0.8 (1.0) | -3.061 | **0.002** | |
| 22 | People look at you oddly because of your appearance | 1.1 (1.2) | 0.8 (1.1) | -1.474 | 0.141 | |
| Negative experiences | | | | | |  |
| 3 | You are not a very animated person | 0.7 (1.0) | 0.8 (0.9) | 0.091 | 0.928 | |
| 4 | Not much of a talker when you are conversing with others | 1.0 (1.1) | 0.6 (0.9) | -2.407 | 0.016 | |
| 8 | Experience few or no emotions at important events | 0.1 (0.6) | 0.3 (0.7) | 1.271 | 0.204 | |
| 16 | No interest to be with other people | 0.9 (1.2) | 0.5 (0.8) | -1.949 | 0.051 | |
| 18 | Lacking in motivation to do things | 1.1 (1.3) | 0.6 (0.8) | -2.255 | 0.024 | |
| 21 | Lacking in energy | 1.5 (1.2) | 0.8 (0.9) | -3.165 | **0.002** | |
| 23 | Mind is empty | 0.7 (1.1) | 0.2 (0.5) | -2.656 | 0.008 | |
| 25 | Spending all your days doing nothing | 1.0 (1.3) | 0.3 (0.7) | -2.857 | 0.004 | |
| 27 | Feelings are lacking in intensity | 1.1 (1.1) | 0.4 (0.9) | -3.228 | **0.001** | |
| 29 | Lacking in spontaneity | 1.0 (1.2) | 0.2 (0.4) | -3.710 | **<0.001** | |
| 32 | Emotions are blunted | 0.8 (1.1) | 0.1 (0.3) | -3.287 | **0.001** | |
| 35 | Neglecting your appearance/personal hygiene | 0.7 (1.1) | 0.3 (0.8) | -1.788 | 0.074 | |
| 36 | Never get things done | 0.9 (1.2) | 0.5 (0.9) | -1.369 | 0.171 | |
| 37 | Have only few hobbies or interests | 1.0 (1.2) | 1.0 (1.0) | -0.140 | 0.889 | |
| Depressive experiences | |  |  |  |  | |
| 1 | Feel sad | 1.4 (1.1) | 1.0 (0.9) | -1.961 | 0.050 | |
| 9 | Feel pessimistic about everything | 1.0 (1.1) | 1.3 (1.2) | 1.163 | 0.245 | |
| 12 | There is no future for you | 0.9 (1.2) | 0.8 (1.1) | -0.530 | 0.596 | |
| 14 | You do not want to live anymore | 0.9 (1.3) | 0.4 (0.8) | -2.100 | 0.036 | |
| 19 | Cry about nothing | 0.7 (1.1) | 0.5 (0.9) | -0.995 | 0.320 | |
| 38 | Feel guilty | 1.1 (1.2) | 0.8 (1.1) | -1.231 | 0.218 | |
| 39 | Feel like a failure | 1.2 (1.3) | 1.0 (1.2) | -0.895 | 0.371 | |
| 40 | Feel tense | 1.2 (1.2) | 0.9 (1.0) | -1.322 | 0.186 | |

A symptom was rated as distressing if distress was at least ‘a bit’ in combination with a frequency of at least ‘sometimes’. Please note: this is in contrast to the calculations of (sub)dimension and total scores, where we dichotomized the item scores. Comparisons between groups were performed using the Wilcoxon signed-rank test.

Bold font denotes statistical significance after Bonferroni correction, considering p < 0.003 as significant for positive experiences (p < 0.05/20 tests), p < 0.004 for negative experiences (p < 0.05/14 tests) and p < 0.006 for depressive experiences (p < 0.05/8 tests).
